# *Ziziphus spina‐christi* (L.) Leaf Extract Improves Growth, Feed Utilization, Digestive Enzymes and Blood Metabolites in Broiler Chickens

**DOI:** 10.1002/vms3.70641

**Published:** 2025-10-15

**Authors:** Fayiz M. Reda, Hassan A. Rudayni, Hemat K. Mahmoud, Nahed A. El‐Shall, Abdullah S. Alawam, Ahmed A. Allam, Mayada R. Farag, Mahmoud Alagawany, Most Khairunnesa

**Affiliations:** ^1^ Poultry Department Agriculture Faculty Zagazig University Zagazig Egypt; ^2^ Department of Biology College of Science Imam Mohammad Ibn Saud Islamic University (IMSIU) Riyadh Saudi Arabia; ^3^ Department of Animal Production Faculty of Agriculture Zagazig University Zagazig Egypt; ^4^ Department Poultry and Fish Diseases Faculty of Veterinary Medicine Alexandria University Alexandria Egypt; ^5^ Forensic Medicine and Toxicology Department Faculty of Veterinary Medicine Zagazig University Zagazig Egypt; ^6^ Department of Dairy and Poultry Science Faculty of Veterinary Medicine and Animal Science Gazipur Agricultural University Gazipur Bangladesh

**Keywords:** blood chemistry, chickens, immunity, intestinal enzymes, performance, *Ziziphus spina‐christi*

## Abstract

**Background:**

*Ziziphus spina‐christi* has been a part of traditional medicine for centuries in North Africa and the Middle East. Numerous secondary metabolites have been isolated from this plant, such as alkaloids, flavonoids, polyphenols, vitamin C and bioactive polysaccharides. Therefore, using *Z. spina‐christi* leaf extract (ZSLE) is one of the recommendations for improving poultry performance and health.

**Objectives:**

The goal of this study was to explore the influences of ZSLE as a feed additive on broiler growth, carcass traits, digestive enzymes, immunological and antioxidant parameters, lipid profile and blood metabolites.

**Methods:**

A total of 250 one‐week‐old Arbor Acres broilers were distributed into 5 groups; each group contained 5 replicates with 10 birds each. The treatments were (1) basal diet (no additive, ZSLE‐0), (2) basal diet + 2 g ZSLE/kg (ZSLE‐2), (3) basal diet + 4 g ZSLE/kg (ZSLE‐4), (4) basal diet + 6 g ZSLE/kg (ZSLE‐6) and (5) basal diet + 8 g ZSLE/kg diet (ZSLE‐8).

**Results:**

*Z. spina‐christi* reduced feed intake while improving body weight gain and feed conversion ratio (FCR) (*p* < 0.05). During the 3–6 and 1–6 weeks, birds fed ZSLE‐4 improved FCR compared with the other birds (*p* < 0.05). Compared to ZSLE‐0, ZSLE‐6 and ZSLE‐8 had substantially greater gizzard and giblet percentages. However, ZSLE had no effect on carcass, dressing, liver or heart percentages. Birds fed ZSLE‐4, ZSLE‐6 and ZSLE‐8 showed increases in amylase, lipase, protease, haemoglobin % (Hb), red blood cells (RBCs) and white blood cells (WBCs) count (*p* < 0.05). ZSLE supplementation had no effect on aspartate aminotransferase (AST) (*p* > 0.05), whereas ZSLE‐4 and ZSLE‐6 decreased alanine aminotransferase (ALT) levels (*p* < 0.05). All ZSLE‐supplemented groups showed improvements in total protein, globulin and lipid profiles. ZSLE significantly decreased cholesterol, low‐density lipoprotein (LDL) and LDL/high‐density lipoprotein (HDL) ratio. HDL was increased in ZSLE‐4, ZSLE‐6 and ZSLE‐8 (*p* < 0.05). Birds fed ZSLE‐6 and ZSLE‐8 improved the immunoglobulins (IgA, IgY and IgM), bursa of Fabricius%, thymus% and spleen% compared with the other birds (*p* < 0.05). Additionally, lysozyme and C3 showed a significant linear influence. All ZSLE‐supplemented groups showed low blood glucose, creatinine, uric acid and malondialdehyde (MDA) (*p* < 0.05). ZSLE improved antioxidant capacity, and ZSLE‐6 and ZSLE‐8 showed the highest reduced glutathione (GSH), catalase (CAT) and total antioxidant capacity (TAC) levels (*p* < 0.05).

**Conclusion:**

Collectively, ZSLE exhibits growth‐promoting, immunostimulant, antioxidant and hypolipidemic properties and can be employed as a natural extract in the diet of broiler chickens.

## Graphical abstract

*Ziziphus spina‐christi* (ZSC) has been a part of traditional medicine for centuries in North Africa and the Middle East, and numerous secondary metabolites have been isolated from this plant. So, the current study investigated the impact of ZSLE, a novel feed additive with varying amounts, and the results indicated that ZSL extract exhibits growth‐promoting, immunostimulant, antioxidant and hypolipidemic properties and can be employed as a natural extract in the diet of broiler chickens.

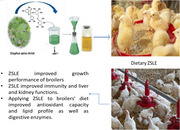

## 1 Introduction

The use of synthetic compounds and antibiotics has been a problem in poultry production due to their residues and resistance in bird tissues, respectively (Li et al. 2023; Durrani et al. 2024). Consequently, alternatives, such as herbal medicines, have been thoroughly investigated for their potential effects on immunological regulation, growth stimulation and antioxidants (Akhtar et al. 2023; Ali et al. 2023; Elzaiat et al. 2024; Alharbi 2024a). *Ziziphus spina‐christi (Christ's Thorn Jujube, (Sidr))* is one of about 100 species of flowering trees, genus *Ziziphus*, family Rhamnaceae, and grows in tropical climates (Mariod et al. 2017). It has been a part of traditional medicine for centuries in North Africa and the Middle East (Mariod et al. 2017). Numerous secondary metabolites have been isolated from this plant and chemically identified, including peptides, cyclopeptide alkaloids, flavonoids, sterols, tannins, polyphenols, botulinic acid, saponins, glycosides, pentacyclic triterpenes, vitamin C and bioactive polysaccharides (Basyony et al. 2017; Cadi et al. 2020; Sakna et al. 2019; Shahat et al. 2001). The most frequently reported chemicals in *Z. spina‐christi* leaves were polyphenols and flavonoids, which accounted for 66 of the 193 compounds identified from various plant sections (Abdulrahman et al. 2022). Sugars, including lactose, glucose, galactose, arabinose, xylose and rhamnose, are also present in the leaves. These secondary metabolites significantly improved the potential therapeutic uses of *Z. spina‐christi* by exhibiting strong anti‐inflammatory (Kadioglu et al. 2016), growth‐promoting (Bilel et al. 2023), antibacterial (Abdulqahar et al. 2023), antiviral (Abdulqahar et al. 2023), antiparasitic, antifungal (Bilel et al. 2023), anticancer (Abdulqahar et al. 2023; Alharbi 2024a), hypolipidemic (El‐Kholy et al. 2024), immunomodulatory (El‐Kholy et al. 2024) and antioxidant (Alharbi 2024b) properties. The leaves of *Z. spina‐christi* were tested as natural growth promoters in growing rabbits (10 and 20 g/kg diet) (Basyony et al. 2017) and developing lambs (15 g/kg DM) (El‐Sheikh et al. 2018). The findings strongly implied that *Z. spina‐christi* leaves might be fed as natural growth promoters without compromising performance. The impact of *Z. spina‐christi* leaf extract (ZSLE) on chickens has been the subject of relatively few studies. In broiler chickens, ZSLE at the level of 10% enhanced production performance, including final weight, weight gain, feed and water consumption, feed conversion ratio (FCR), production index and decreased mortality (Yusup et al. 2021). In a different investigation on laying hens, El‐Kholy et al. (2024) found that dietary *Z. spina‐christi* leaves (7.5 mL/kg) had an antioxidant effect and improved egg quality and laying performance under heat stress. The current study investigated the impact of ZSLE, a novel feed additive with varying amounts (0–8 g/kg diet), on growth, carcass traits, digestive enzymes, immunological and antioxidant parameters, lipid profile and blood metabolites in Arbor Acres broilers.

## 2 Materials and Methods

### 2.1 Animal Husbandry and Experimental Design

A total of 250 one‐week‐old Arbor Acres broilers were distributed into 5 groups; each group contained 5 replicates with 10 birds each. The treatments were (1) basal diet (no additive, ZSLE‐0), (2) basal diet + 2 g ZSLE/kg (ZSLE‐2), (3) basal diet + 4 g ZSLE/kg (ZSLE‐4), (4) basal diet + 6 g ZSLE/kg (ZSLE‐6) and (5) basal diet + 8 g ZSLE/kg diet (ZSLE‐8). A completely randomized design was used to assign the chicks. As shown in Table 1, the experimental food was formulated to fulfil the requirements of the Arbor Acres breed. While maintaining the humidity at about 60% throughout the experiment, the rearing temperature gradually lowered from 33°C + 1°C for the first 3 days to 24°C by the end of the third week. Beginning on Day 0, the lighting regimen consisted of 23 h of light, which decreased to 18 h on Day 3 and then 17 h on Day 4 and beyond. The birds were kept in standard cages measuring 120 by 100 cm, and they were given unrestricted access to feed and water during the trial (starter 1–3 weeks, finisher 3–6 weeks). All birds were kept under the same management conditions. Vaccination and medical program were done according to the different stages of age under the supervision of a veterinarian.

**TABLE 1:** Composition and chemical calculation of the experimental diets (starter and finisher diets).

Items	Starter (1–3 weeks)	Finisher (4–6 weeks)
Ingredients (%)		
Corn	57.9	62.00
Soybean meal 44% CP	33	26.20
Soybean oil	1.50	3.00
Corn gluten 62% CP	3.5	4.80
Mono calcium phosphate	1.70	1.55
Limestone	1.30	1.22
Vit–min premix^a^	0.30	0.30
NaCl	0.30	0.30
DL methionine	0.10	0.05
l‐Lysine	0.05	0.28
Choline chloride 60%	0.05	0.30
Total	100	100
Calculated and determined analysis^b^ (%)		
Crude protein	23.00	20.10
Crude protein (analysed)^c^	23.02	19.90
Crude fat (analysed)^c^	4.11	5.73
Metabolizable energy kcal/kg diet	2900	3100
Calcium	1.00	0.95
Phosphorus (available)	0.48	0.41
Total lysine	1.40	1.20
Total methionine + cysteine	0.92	0.72
Crude fibre	3.43	3.26

^a^ Growth vitamin and mineral premix. Each 2.5 kg consists of Vit A, 12,000,000 IU; Vit D3, 2,000,000 IU; Vit. E., 10 g; Vit k3, 2 g; Vit B1, 1000 mg; Vit B2, 49 g; Vit B6, 105 g; Vit B12, 10 mg; pantothenic acid, 10 g; niacin, 20 g; folic acid, 1000 mg; biotin, 50 g; choline chloride, 500 mg, Fe, 30 g; Mn, 40 g; Cu, 3 g; Co, 200 mg; Si, 100 mg and Zn, 45 g.

^b^ Calculated according to National Research Council (NRC) (1994).

^c^ Determined according to AOAC (2006).

### 2.2 Growth, Carcass Characteristics and Immune Organ

The body weight (BW) (g) and weight gain (g/day) of broiler chickens were calculated by recording their individual weights at Weeks 1, 3 and 6. Throughout the trial, feed intake (g) was recorded and converted to g feed/g gain. At 42 days, five birds/group were chosen at random and slaughtered to evaluate the carcass characteristics (Reda et al. 2025). Weighing each edible component, including the giblets (liver, heart and gizzard) and the eviscerated carcass, allowed us to calculate the percentage of pre‐slaughter weight. Dressing % = (carcass weight + giblets weight)/pre‐slaughter weight × 100. The carcasses were divided into several sections, such as the thighs and breast with bones and skin, after being chilled at 1°C for 12 h. Until their analysis, these carcass sections were kept at −20°C. Moreover, immune organs, including the spleen, thymus and bursa of Fabricius, were weighed to calculate their percentages.

### 2.3 Haematological and Blood Biochemistry Analysis

At 6 weeks of age, five randomly chosen birds per group were sedated with a ketamine–xylazine cocktail (2:1), weighed and slaughtered with a sharp knife. Their blood was then collected in sterile tubes containing EDTA. To combine the blood with the anticoagulant, the test tubes were then gently shaken. Haematological parameters were assessed using whole blood samples to measure white blood cells (WBCs, 10^3^/µL), red blood cells (RBCs, 10^6^/µL), haemoglobin (Hb, g/dL) and glucose (mg/dL). The analyses were conducted in accordance with the methodology outlined by Jain (1986) using an automated cell counter (model XE‐2100, Sysmex, Kobe, Japan). To extract the separated plasma, the whole blood was centrifuged for 20 min at 3000 rpm. The plasma was then stored at −20°C until analysis. The concentrations of albumin (ALB—g/dL) and total protein (TP—g/dL) were measured using the calorimetric method described by Armstrong and Carr (1964). To calculate the globulin concentrations (g/dL), the ALB concentrations were deducted from the TP concentrations.

Commercial kits were utilized to evaluate urea, uric acid and creatinine, as well as the liver function‐indicating enzymes (AST: aspartate aminotransferase and ALT: alanine aminotransferase), as indicated by the manufacturer (Labtest Diagnóstica S.A., Lagoa Santa, Brazil). According to Allain et al. (1974), total cholesterol and triglycerides were assessed. Friedewald et al. (1972) report low‐density lipoprotein (LDL) and high‐intensity lipoprotein (HDL) quantities. Furthermore, measured using the methodology described by Bianchi et al. (1995) were the immunoglobulins IgA, IgM and IgY (mg/dL). Lysozyme activity was ascertained by use of the lysozyme's capacity to lyse *Micrococcus lysodeikticus* in 1% agarose at 37°C for 18 h and in the presence of hen egg white lysozyme solution (20 mg/mL). Using commercially sold chicken‐specific ELISA kits in conformity with the policies of the respective firms, complement (C3) was measured (MyBioSource Inc., CA, USA; Bethyl Labs, Montgomery, TX, USA). Based on Koracevic (2001), the total antioxidant capacity (TAC) was calculated. Spectrophotometer measurements of superoxide dismutase (SOD), lowered glutathione (GSH) and catalase (CAT) in line with (Nishikimi et al. 1972). The approach suggested by Mihara and Uchiyama (1978) was followed to measure malondialdehyde (MDA).

### 2.4 Digestive Enzymes

The bird's ileum was dissected from the Meckel's diverticulum up to 2 cm above the ileocaecal junction, and the ileal contents were collected in a sterile specimen vial with a screw top. The activity of lipase, protease and amylase enzymes in each bird's ileum was assessed at the end of the study using the technique of Najafi et al. (2006).

### 2.5 Statistical Analysis

All the statistical assessments were carried out using SAS (SAS 2001) and a one‐way analysis of variance (ANOVA) (SAS software, Cary, NC, USA). The statistical model applied was *Y_ij_* = *μ* + *T_i_* + *e_ij_*, where *Y_ij_* is an observation; *μ* is the overall mean; *T_i_* is the fixed effect of treatments; and *e_ij_* is the random error. All data on growth performance, carcass traits, blood biochemistry, immune responses, digestive enzyme activities and antioxidant parameters were analysed using Tukey's multiple comparison test at a significance level of *p* < 0.05. Orthogonal polynomial contrasts were used to test the significance (linear and quadratic) of the gradual dietary levels *of* ZSLE.

## 3 Results

### 3.1 Growth Performance and Carcass Traits

Supplementing broiler chickens with various dosages of ZSLE at 3 and 6 weeks of age resulted in a substantial improvement in BW as compared to non‐supplemented birds (*p* < 0.05). However, ZSLE‐2 demonstrated a lower body weight gain (BWG) and BW than ZSLE‐4, ZSLE‐6 and ZSLE‐8 (*p* < 0.05). However, ZSLE‐4, ZSLE‐6 and ZSLE‐8 showed comparable results (*p* > 0.05). At 1–6 weeks, all ZSLE‐supplemented groups had lower FI than ZSLE‐0 (*p* < 0.05). ZSLE‐6 had the lowest FI at 1–6 weeks, which was statistically similar to ZSLE‐4 and ZSLE‐8 (*p* > 0.05). All tested doses of ZSLE improved FCR during the study (*p* < 0.05), with ZSLE‐6 having the lowest FCR at both 3–6 and 1–6 weeks (Table 2).

**TABLE 2:** Growth performance of broilers as affected by dietary *Ziziphus spina‐christi* leaf extract.

	*Ziziphus spina‐christi* leaf extract (g/kg diet)						*p* value	
Items	0	2	4	6	8	SEM	*L*	*Q*
Body weight (g)								
1 week	145.87	146.23	145.45	146.25	145.50	1.359	0.8724	0.9020
3 week	816.52^c^	848.77^b^	879.40^a^	903.50^a^	907.42^a^	9.010	<0.0001	0.0954
6 week	2011.45^c^	2087.08^b^	2174.70^a^	2233.70^a^	2181.85^a^	19.436	<0.0001	0.0031
Body weight gain (g/day)								
1–3 week	47.90^c^	50.18^b^	52.43^a^	54.09^a^	54.42^a^	0.695	<0.0001	0.1216
3–6 week	56.90^c^	58.97^bc^	61.68^ab^	63.34^a^	60.69^ab^	1.062	0.0068	0.0304
1–6 week	53.30^c^	55.45^b^	57.98^a^	59.64^a^	58.18^a^	0.526	<0.0001	0.0022
Feed intake (g/day)								
1–3 week	79.98	78.04	78.77	77.78	76.99	1.233	0.1969	0.9161
3–6 week	126.57^a^	118.72^ab^	110.12^bc^	109.06^c^	116.14^bc^	2.484	0.0064	0.0052
1–6 week	107.93^a^	102.45^b^	97.58^bc^	96.54^c^	100.48^bc^	1.424	0.0023	0.0039
Feed conversion ratio (g/g)								
1–3 week	1.67^a^	1.56^b^	1.50^bc^	1.44^c^	1.42^c^	0.030	<0.0001	0.1919
3–6 week	2.22^a^	2.01^b^	1.79^cd^	1.72^d^	1.91^bc^	0.050	0.0002	0.0005
1–6 week	2.02^a^	1.85^b^	1.68^cd^	1.62^d^	1.73^c^	0.031	<0.0001	0.0002

*Note*: Means in the same row with different superscript letters are significantly different (*p* < 0.05).

Comparing non‐supplemented controls, ZSLE supplementation did not significantly affect the percentage of carcass, dressing, liver and heart (*p* > 0.05), although numerical increases were observed. However, gizzard and giblet percentage was significantly higher in birds fed ZSLE‐6 and ZSLE‐8 compared to the ZSLE‐0 (*p* < 0.05) (Table 3).

**TABLE 3:** Carcass traits (based on % of live body weight) of broilers as affected by dietary *Ziziphus spina‐christi* leaf extract.

	*Ziziphus spina‐christi* leaf extract (g/kg diet)						*p* value	
Items	0	2	4	6	8	SEM	*L*	*Q*
Carcass %	70.94	71.85	72.13	73.21	73.37	0.895	0.0573	0.8440
Liver %	2.62	2.79	2.73	2.98	2.83	0.133	0.1971	0.5277
Gizzard %	2.00^c^	2.11^bc^	2.33^abc^	2.57^ab^	2.70^a^	0.135	0.0024	0.8871
Heart %	0.71	0.76	0.77	0.82	0.75	0.065	0.5093	0.4409
Giblets %	5.34^c^	5.66^bc^	5.84^abc^	6.37^a^	6.28^ab^	0.149	0.0015	0.5374
Dressing %	76.27	77.51	77.96	79.58	79.66	0.889	0.0123	0.7447

*Note*: Means in the same row with no superscript letters are not significantly different (*p* < 0.05).

### 3.2 Haematology, Blood Protein and Liver and Kidney Functions

Haemoglobin increased linearly with increasing ZSLE dose, with doses of 4, 6 and 8 g/kg showing the greatest effect (*p* < 0.05), 11.88, 12.45 and 13.02 g/dL, respectively. All ZSLE‐supplemented groups had significantly higher RBC counts (*p* = 0.0016). WBC counts increased with ZSLE‐8 compared to ZSLE‐0 (*p* = 0.0078). When compared to controls, ZSLE supplementation dramatically lowered glucose levels (*p* = 0.0001), decreasing further with higher doses (Table 4).

**TABLE 4:** Haematology of broilers as affected by dietary *Ziziphus spina‐christi* leaf extract.

	*Ziziphus spina‐christi* leaf extract (g/kg diet)						*p* value	
Items	0	2	4	6	8	SEM	*L*	*Q*
Haemoglobin (g/dL)	10.76^c^	11.07^c^	11.88^b^	12.45^ab^	13.02^a^	0.260	<0.0001	0.7792
RBCs (10^6^/µL)	2.44^b^	2.78^a^	2.72^a^	3.01^a^	2.96^a^	0.090	0.0016	0.2251
WBCs (10^3^/µL)	25.65^bc^	25.05^c^	26.55^abc^	29.39^ab^	30.08^a^	1.219	0.0078	0.4051
Glucose (mg/dL)	321.05^a^	292.43^b^	264.52^c^	236.84^d^	253.47^cd^	4.618	<0.0001	0.0338

*Note*: Means in the same row with different superscript letters are significantly different (*p* < 0.05).

Birds with ZSLE‐4, ZSLE‐6 and ZSLE‐8 had higher levels of TP and globulin (GLOB) than birds without supplements (*p* < 0.05). The ZSLE‐6 and ZSLE‐8 produced the highest TP and GLOB levels (*p* < 0.05). There were no statistically significant variations in the ALB and A/G ratios between the experimental groups (*p* > 0.05) (Table 5).

**TABLE 5:** Liver and kidney functions of dietary *Ziziphus spina‐christi* leaf extract.

	*Ziziphus spina‐christi* leaf extract (g/kg diet)						*p* value	
Items	0	2	4	6	8	SEM	*L*	*Q*
TP (g/dL)	3.53^c^	3.76^bc^	4.12^b^	4.63^a^	4.56^a^	0.127	<0.0001	0.4040
ALB (g/dL)	2.04	2.14	2.26	2.42	2.33	0.118	0.0487	0.4759
GLOB (g/dL)	1.49^c^	1.62^bc^	1.85^b^	2.21^a^	2.23^a^	0.087	<0.0001	0.7446
A/G (%)	1.40	1.33	1.22	1.11	1.05	0.081	0.0164	0.9725
ALT (IU/L)	20.86^a^	17.32^ab^	12.12^c^	13.79^bc^	17.96^ab^	1.302	0.0519	0.0012
AST (IU/L)	85.74	78.37	85.64	75.47	77.05	3.724	0.1554	0.9767
Uric acid (mg/dL)	7.23^a^	5.52^bc^	5.73^b^	4.73^bc^	4.35^c^	0.344	0.0002	0.3143
Urea (mg/dL)	1.81	2.02	1.49	1.62	1.65	0.120	0.0962	0.5441
Creatinine (mg/dL)	0.96^a^	0.74^b^	0.68^bc^	0.54^c^	0.72^b^	0.047	0.0013	0.0028

*Note*: Means in the same row with different superscript letters are significantly different (*p* < 0.05).

Abbreviations: A/G, albumin/globulin ratio; ALB, albumin; ALT, alanine aminotransferase; AST, aspartate aminotransferase; GLOB, globulin; TP, total protein.

ZSLE‐4 and ZSLE‐6 showed a significant (quadratic, *p* = 0.0012) decrease in ALT levels, although ZSLE‐2 and ZSLE‐8 showed a numerical decline. ZSLE supplementation, however, had no discernible impact on the AST level (Table 5). Interestingly, all ZSLE dosages significantly reduced uric acid (linear, *p* = 0.0002) and creatinine (linear and quadratic, *p* = 0.0013 and 0.0028, respectively) levels (Table 5).

### 3.3 Lipid Profile and Digestive Enzymes

The ZSLE‐supplemented groups showed an improvement in their lipid profile (Table 6). Although TG and VLDL fell in ZSLE‐4 and ZSLE‐6 (*p* < 0.05), TC, LDL and the LDL/HDL ratio statistically decreased in all ZSLE‐supplemented groups (*p* < 0.05) compared to the control. ZSLE‐4, ZSLE‐6 and ZSLE‐8 had higher HDL (*p* < 0.05) than the control.

**TABLE 6:** Lipid profile of dietary *Ziziphus spina‐christi* leaf extract.

	*Ziziphus spina‐christi* leaf extract (g/kg diet)						*p* value	
Items	0	2	4	6	8	SEM	*L*	*Q*
TC (mg/dL)	158.59^a^	136.57^b^	138.26^b^	132.51^bc^	123.88^c^	3.395	<0.0001	0.1923
TG (mg/dL)	65.12^a^	56.48^ab^	48.04^b^	39.23^c^	59.33^a^	2.718	0.0078	0.0002
HDL (mg/dL)	33.44^c^	34.98^c^	51.61^b^	65.45^a^	56.69^ab^	2.672	<0.0001	0.0496
LDL (mg/dL)	112.12^a^	90.30^b^	77.04^b^	59.21^c^	55.32^c^	4.289	<0.0001	0.0862
VLDL (mg/dL)	13.02^a^	11.30^ab^	9.61^b^	7.84^c^	11.87^a^	0.544	0.0078	0.0002
LDL/HDL ratio	3.39^a^	2.58^b^	1.51^c^	0.92^d^	1.00^c^	0.148	<0.0001	0.0069

*Note*: Means in the same row with different superscript letters are significantly different (*p* < 0.05).

Abbreviations: HDL, high‐density lipoprotein; LDL, low‐density lipoprotein; TC, total cholesterol; TG, triglycerides; VLDL, very low‐density lipoprotein.

Digestive enzyme levels are presented in Table 7, and ZLSE supplementation increased the digestive enzymes. Amylase and lipase activities were significantly higher in ZSLE‐4, ZSLE‐6 and ZSLE‐8 than in ZSLE‐0 and ZSLE‐2 (*p* < 0.05). Protease level also increased at the inclusion level ≥2 g/kg (*p* < 0.05), which shows a dose‐dependent rise with increasing ZSLE supplementation.

**TABLE 7:** Digestive enzymes of dietary *Ziziphus spina‐christi* leaf extract.

	*Ziziphus spina‐christi* leaf extract (g/kg diet)						*p* value	
Items	0	2	4	6	8	SEM	*L*	*Q*
Amylase (μ/L)	36.93^c^	38.59^c^	52.30^b^	61.35^a^	58.07^ab^	2.065	0.0002	0.0969
Lipase (μ/L)	16.91^b^	22.79^b^	30.62^a^	32.52^a^	35.25^a^	1.866	<0.0001	0.1511
Protease (μ/L)	0.81^e^	1.41^d^	2.14^c^	2.85^b^	3.61^a^	0.115	<0.0001	0.4879

*Note*: Means in the same row with different superscript letters are significantly different (*p* < 0.05).

### 3.4 Immune and Antioxidant Parameters

ZSLE supplementation enhanced the percentage of immunological organs in broiler chickens when compared to birds that were not supplemented (Table 8). ZSLE‐4, ZSLE‐6 and ZSLE‐8 showed statistically significant increases in thymus and BF% (*p* < 0.05), but spleen% was statistically increased in ZSLE‐6 and ZSLE‐8 (*p* < 0.05). Lysozyme and C3 levels were increased by increasing ZSLE dose. Likewise, ZSLE‐6 and ZSL‐8 exhibited the highest IgA, IgY and IgM levels compared to non‐supplemented birds (*p* < 0.05) (Table 8).

**TABLE 8:** Immunity of dietary *Ziziphus spina‐christi* leaf extract.

	*Ziziphus spina‐christi* leaf extract (g/kg diet)						*p* value	
Items	0	2	4	6	8	SEM	*L*	*Q*
Immune organs								
Spleen, %	0.12^b^	0.14^b^	0.16^b^	0.22^a^	0.21^a^	0.012	0.0002	0.5494
Thymus, %	0.39^c^	0.44^bc^	0.50^ab^	0.53^ab^	0.55^a^	0.029	0.0020	0.4398
Bursa of Fabricius, %	0.17^c^	0.21^bc^	0.25^ab^	0.27^a^	0.26^a^	0.016	0.0006	0.0752
Immune parameters								
Lysozyme (mg/dL)	0.55^c^	0.68^bc^	0.78^abc^	0.92^ab^	1.03^a^	0.078	0.0008	0.9564
C3 (mg/dL)	31.29^c^	36.82^c^	49.79^b^	57.98^ab^	61.72^a^	2.773	<0.0001	0.4685
IgA (mg/dL)	3.78^c^	4.35^b^	4.67^b^	5.47^a^	5.69^a^	0.158	0.0002	0.7546
IgY (mg/dL)	1.39^d^	1.69^bc^	1.58^cd^	1.89^ab^	2.02^a^	0.074	0.0001	0.7458
IgM (mg/dL)	1.09^d^	1.27^cd^	1.43^bc^	1.81^a^	1.62^ab^	0.084	0.0003	0.1421

*Note*: Means in the same row with different superscript letters are significantly different (*p* < 0.05).

Abbreviations: C3, Complement 3; IgY, IgM and IgA, immunoglobulin Y, M and A.

ZSLE's antioxidant activity is displayed in Table 9. MDA was considerably lower in all ZSLE‐supplemented groups than in the non‐supplemented group (*p* < 0.05), and there was no difference among the supplemented groups (*p* > 0.05). All ZSLE‐supplemented groups showed increases in TAC (*p* < 0.05), GSH (*p* < 0.05) and SOD (*p* > 0.05). Significant increases in CAT were observed in ZSLE‐4, ZSLE‐6 and ZSLE‐8. Among the ZSLE groups, GSH was significantly elevated in ZSLE‐6 (*p* < 0.05), with comparable levels observed in ZSLE‐8. Compared to ZSLE‐2 and ZSLE‐4, ZSLE‐6 and ZSLE‐8 had greater levels of TAC.

**TABLE 9:** Antioxidants of dietary *Ziziphus spina‐christi* leaf extract.

	*Ziziphus spina‐christi* leaf extract (g/kg diet)						*p* value	
Items	0	2	4	6	8	SEM	*L*	*Q*
GSH (mg/dL)	0.20^d^	0.30^c^	0.38^b^	0.48^a^	0.44^ab^	0.021	<0.0001	0.0114
MDA (nmol/mL)	0.42^a^	0.28^b^	0.22^b^	0.24^b^	0.28^b^	0.024	0.0023	0.0008
SOD (U/mL)	0.16	0.18	0.17	0.20	0.18	0.019	0.3820	0.5612
CAT (mg/dL)	0.16^d^	0.24^cd^	0.25^bc^	0.33^ab^	0.36^a^	0.026	0.0002	0.7164
TAC (ng/mL)	0.11^d^	0.22^c^	0.30^b^	0.34^a^	0.35^a^	0.012	<0.0001	0.0006

*Note*: Means in the same row with different superscript letters are significantly different (*p* < 0.05).

Abbreviations: CAT, catalase; GSH, reduced glutathione; MDA, malondialdehyde; SOD, superoxide dismutase; TAC, total antioxidant capacity.

## 4 Discussion

All tested ZSLE dosages had a beneficial effect on the growth performance of broiler chicks (increased BW, BWG and FCR with decreased feed intake—FI). It was found that the improvement in BW, BWG and FCR started 2 weeks after ZSLE supplementation (1–3 weeks of age). Although there was no statistically significant decrease in FI at 1–3 weeks, ZSLE‐supplemented groups showed a substantial improvement in FCR throughout this time, with significantly greater effects observed at ≥4 g of ZSLE/kg. Thus, the improvement in FCR in ZSLE‐supplemented groups is ascribed to additional beneficial effects on the body rather than a decrease in FI. The effects of varying concentrations (10%, 15% and 20%) of ZSLE in drinking water on broiler chicken productivity and mortality were examined by Yusup et al. (2021). They found that ZSLE in drinking water increased BWG, final BW, FCR and water consumption and decreased mortality rate. In the same study, FI was increased by ZSLE in drinking water. Similar to our findings, aqueous ZSLE addition to dual‐purpose laying hens’ meals (2.5, 5.0 and 7.5 mL/kg) under subtropical farming significantly reduced FI while increasing BW, weight gain and FCR (El‐Kholy et al. 2024). *Z. spina‐christi* contains polyphenols, saponins, tannins and sterols, such as ß‐sitosterol, terpenoids, phytosterols and triterpenoids, in addition to alkaloids, flavonoids and glycosides. These active ingredients have antibacterial, antioxidant and digestive enzyme‐stimulating properties (Ibraheem 2017). ZSLE‐4, ZSLE‐6 and ZSLE‐8 exhibited a considerable increase in digestive enzymes, with a linear rise in lipase and protease. Furthermore, *Z. spina‐christi* leaves significantly reduced the number of bacteria overall as well as the counts of *Escherichia coli* and *Clostridium* (Abduljawad 2020). This effect might be explained by the high concentration of condensed tannins in *Ziziphus* leaves. ZSLE may therefore be used to increase broiler growth and enhance FCR by enhancing nutrient digestibility, absorption and utilization and regulating beneficial microbes in the intestines.

MDA is frequently employed as a marker for lipid peroxidation damage caused by free radicals, whereas CAT, SOD, TAC and GSH are crucial elements of the antioxidant defence system. In the present study, ZSLE significantly reduced MDA and increased CAT, TAC and GSH, hence inducing antioxidant activity. These findings are in accordance with Abdel‐Wahhab et al. (2007), Abduljawad (2020) and Amin and Mahmoud‐Ghoneim (2009). The high quantities of triterpene, tocopherols and polyphenols were thought to be responsible for this activity (Hussein 2019; Sakna et al. 2019). In addition to flavonoids (Dawood et al. 2024; Milad et al. 2023), they are believed to be the most powerful dietary antioxidants (Anderson 1980).

Flavonoids have been implicated in immunological response through cytokine regulation and TNF‐α suppression. In addition, flavonoids have the ability to restore lymphocyte proliferation and stop apoptosis (Nair et al. 2006). Accordingly, we found that large doses of ZSLE (6 and 8 g/kg) markedly raised WBC count, lysozyme, Complement 3, IgA, IgM and IgY levels, as well as immunological organ%. Similarly, *Z. spina‐christi* leaf supplementation significantly raised the serum levels of IgA, IgG and IgM in rabbits, according to Abduljawad (2020). Additionally, in immune‐compromised rats, *Z. spina christi* extracts increased the levels of tumour necrosis factor, Complement 3, Complement 4, interferon‐gamma and total leukocyte counts (Milad et al. 2023).

When broiler chickens were supplemented with ZSLE, their blood lipid profile significantly improved (HDL increased, and cholesterol, triglycerides, LDL, VLDL and the LDL/HDL ratio decreased). This effect of hypolipidemia was consistent with the findings of Abdel‐Wahhab et al. (2007). These results could improve the quality of broiler meat by lowering its fat content.

Glucose is the primary carbohydrate in birds’ blood and can be used as an energy source by the cells. Glucagon, insulin and other pancreatic hormones regulate its concentration (Scanes 2015). A common biological evaluation index in research assessing how dietary components affect health problems is blood glucose concentration (Kawasaki et al. 2020). The current investigation found that ASLE had a hypoglycaemic impact, resulting in a marked drop in blood glucose levels. Similarly, a butanol extract of *Z. spina‐christi* leaves was found to have an antihyperglycaemic action in both diabetic and non‐diabetic rats (Abdel‐Zaher et al. 2005). They attributed it to the insulinotropic effect, which is the intensification of glucose‐induced insulin production. ZSLE's saponin and polyphenol concentrations may both contribute to this insulinotropic action (Michel et al. 2011). However, broiler deaths or health issues such as spiking mortality syndrome can result from severe hypoglycaemia (Burns et al. 2002). There were no discernible adverse consequences of hypoglycaemia in this investigation. In order to determine the extent of ZSLE‐induced hypoglycaemia and its impact on broiler health, this effect should be further examined by using greater dosages of ZSLE in the diet of broiler chickens.

Toxins and their metabolites are mostly dealt with by the liver and kidney through biotransformation, detoxification and excretion. They are hence susceptible to hazardous harm (Mathew 1992; Delaney et al. 1998). Furthermore, some substances have negative haematologic consequences when administered at toxic or therapeutic dosages (Abdel‐Zaher et al. 2005) such as reduction in the number of blood cells and anomalies in their structure and function (Max 1996). According to measured biochemicals, the results of this study suggest that supplementing broiler chickens with ZSLE did not cause any toxicity or adverse effects on their liver, kidney or haematology.

## 5 Conclusions

ZSLE improved growth performance at certain inclusion levels and had positive effects on digestive enzymes, haematology and lipid profile. The addition of ZSLE to broiler chickens’ feed improved their growth performance (improved BWG and FCR and decreased feed intake). Higher levels of TP, globulin and digestive enzymes were observed in the *Z. spina‐christi* leaf at 4–8 g/kg diet in comparison to the control group. High dosages of *Z. spina‐christi* leaf (6 and 8 g/kg) considerably improved immunological parameters and enhanced carcass characteristics (gizzard % and giblets %). Additionally, *Z. spina‐christi* leaf has antioxidant, hypoglycaemic and hypolipidemic effects. *Z. spina‐christi* leaf was safe and had no harmful effects on the kidney, liver or haematological system. *Z. spina‐christi* leaf can therefore be employed as a natural antioxidant, immune‐stimulating and growth‐promoting agent in broiler chickens. These findings suggest that ZSLE could be considered a natural growth promoter and health enhancer in broiler diets; however, further research on its economic feasibility and safety at higher inclusion levels is required.

## Author Contributions

Fayiz M. Reda Abdullah S. Alawam, Hemat K. Mahmoud, Nahed A. El‐Shall, Hassan A. Rudayni, Ahmed A. Allam, Mayada R. Farag, Mahmoud Alagawany and Most Khairunnesa were involved in conceptualization, data curation, formal analysis, investigation, methodology, resources, validation, visualization, roles/writing of the original draft, writing review and editing.

## Ethics Statement

ZSLE was obtained from the Agricultural Microbiology Department, Faculty of Agriculture, Zagazig University, Egypt. During the experimental processes, the Local Experimental Animal Care Committee's developed protocols were followed. The ethical approval code is ZU–IACUC/2/F/313/2023.

## Conflicts of Interest

The authors declare no conflicts of interest.

## Peer Review

The peer review history for this article is available at https://www.webofscience.com/api/gateway/wos/peer‐review/10.1002/vms3.70641.

## Data Availability

The data in this study can be obtained upon request from the relevant authors.
